# Supplementary material for: A transportome-scale amiRNA-based screen identifies redundant roles of Arabidopsis ABCB6 and ABCB20 in auxin transport
Source: Nat Commun. 2018 Oct 11;9:4204. doi: 10.1038/s41467-018-06410-y (PMC6182007; doi:10.1038/s41467-018-06410-y)
Supplement: Supplementary file 1 — Supplementary Information [file 41467_2018_6410_MOESM1_ESM.pdf]

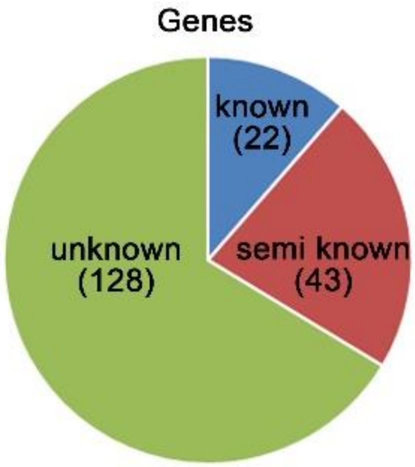

Supplementary Figure 1. Analysis of identified amiRNA lines phenotypes according to putative target genes loss-of-function phenotype found in published literature. The number of identified genes in our study that are known, semi known and unknown compared to the published literature. Semi known genes are defined for target genes that were reported to show a loss-of-function phenotype that is different from the phenotype observed in this study (different developmental stage and/or different target combination).

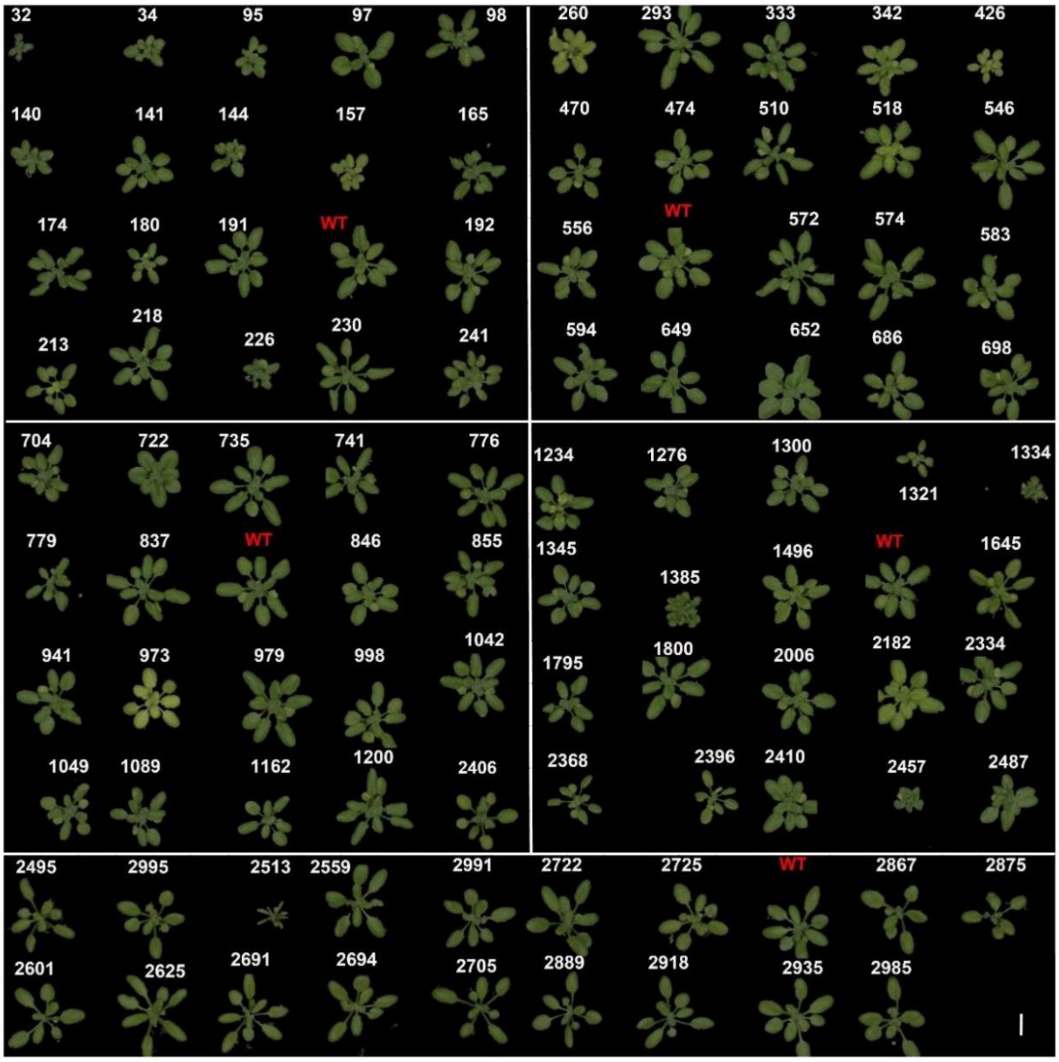

Supplementary Figure 2. Representative shoot phenotypes of 25-day-old amiRNA lines from the transporter library. Numbers correspond to amiRNA lines (Supplementary Table 1). Scale bar, 1 cm.

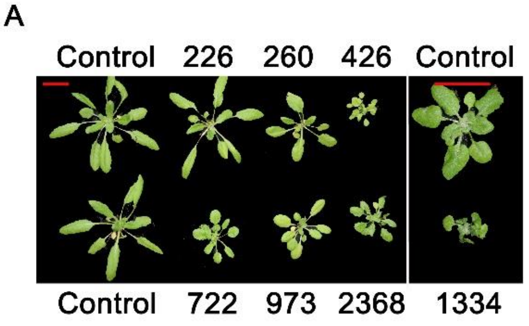

B

Line	Ratio	Percentage(%)
226	0/48	0
260	37/45	82
426	39/45	87
722	27/52	52
973	34/48	71
2368	35/44	80
1334	45/52	86

Supplementary Figure 3. Transportome amiRNA reproducibility and variation. Seven representative amiRNA lines were re-cloned and transformed into Col-0. **(A)** Shoot phenotypes of 30-day-old amiRNA lines grown in soil. Scale bar, 1 cm. **(B)** Percentage of independent transformation lines presenting phenotypes. All lines are compared to an empty vector transformation control in T_1 generation. Line 1334 is in T_2 generation compared to its respective control.

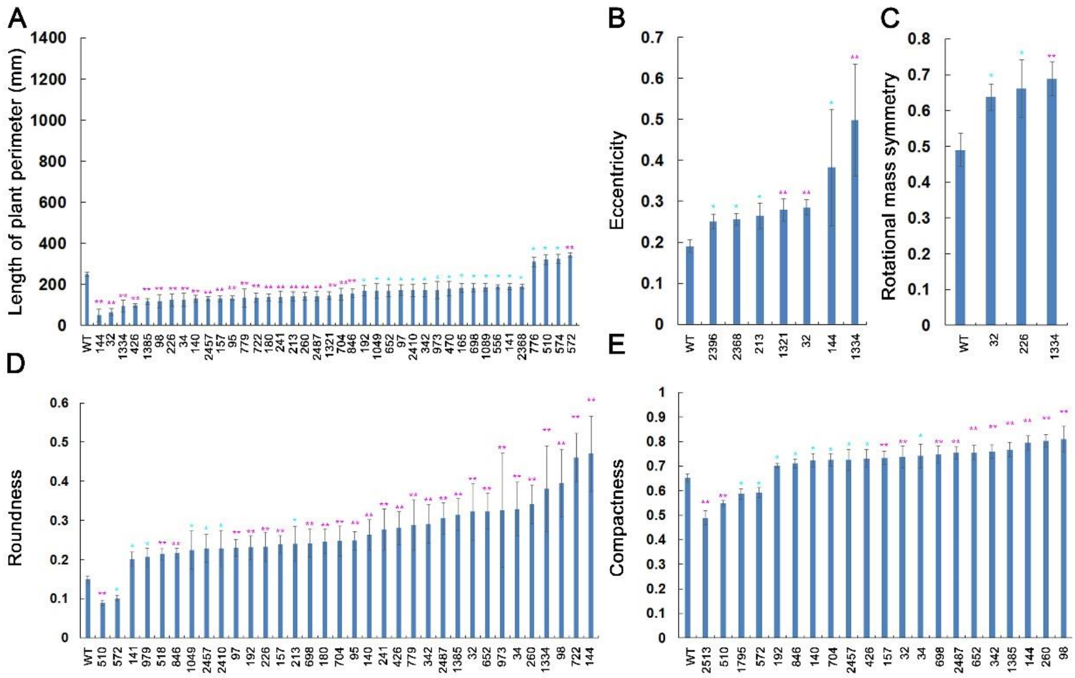

Supplementary Figure 4. Morphological parameters of 25-day-old amiRNA lines. All parameters were assayed using a phenomics system. Presented are statistically significant amiRNA phenotypes. X-axis denotes amiRNA lines. **(A)** Length of rosette perimeter. **(B)** Eccentricity of plant perimeter, describing the extent of rosette deviates from being circular. **(C)** Rotational mass symmetry of plant perimeter. **(D)** Roundness (index of rosette shape). **(E)** Plant compactness (parameter includes petiole length). The higher the compactness, the shorter are the petioles and the larger are leaf blades. For all the panels above, $n = 5$ plants. Differences are significant at $P < 0.05$ (*) and $P < 0.01$ (**) by Student's t-test.

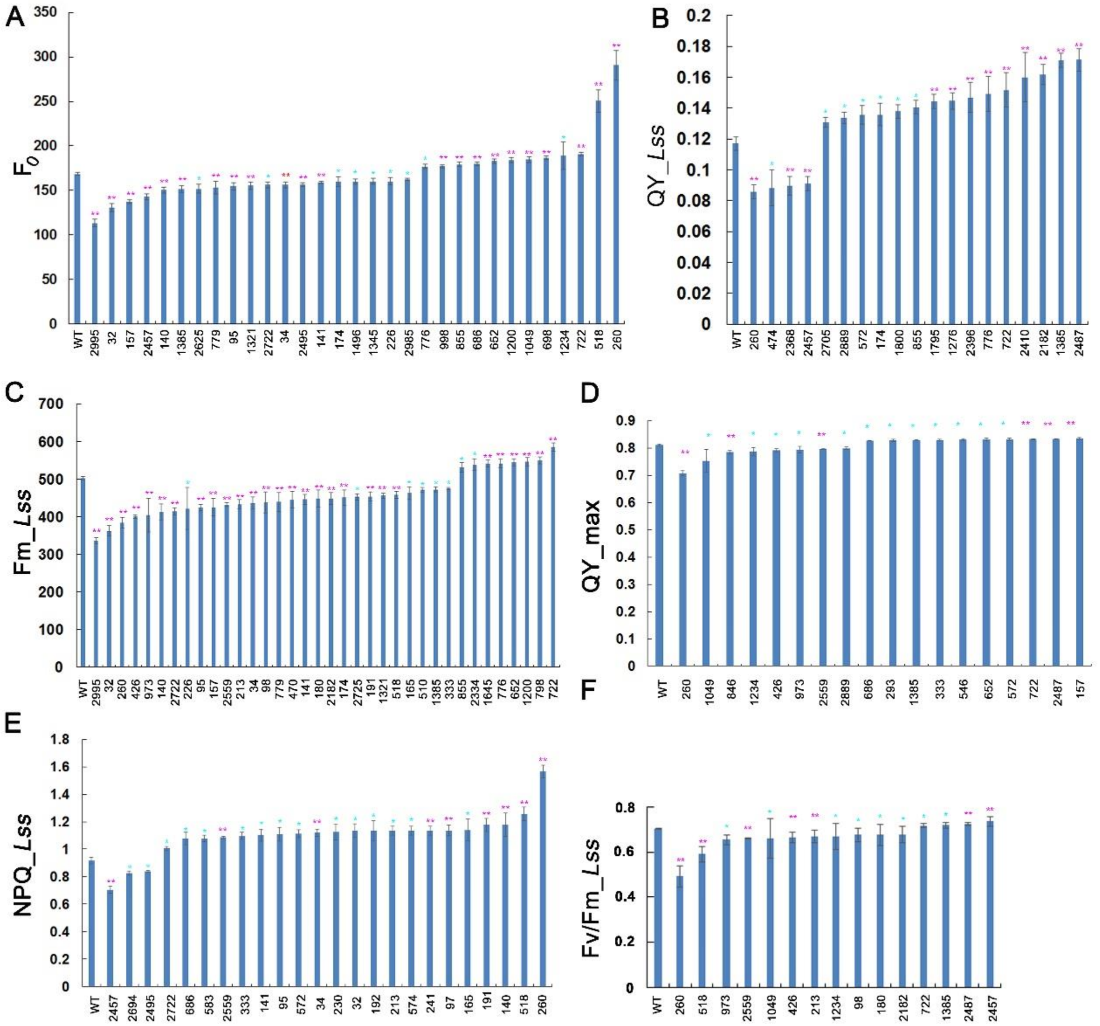

Supplementary Figure 5. Photosynthesis parameters of 25-day-old amiRNA lines. All parameters were assayed using a phenomics system. Presented are statistically significant amiRNA phenotypes. X-axis denotes amiRNA lines. **(A)** F_0, minimum fluorescence in dark-adapted state. **(B)** *QY_Lss*, steady-state PSII quantum yield in light. **(C)** *Fm_Lss*, a measured index, describing the steady-state maximum fluorescence in light. Non-photochemical quenching at maximum (NPQ max). **(D)** *QY_max*, calculated by *Fv*/*Fm*, maximum PSII quantum yield in dark-adapted state. **(E)** *NPQ_Lss*, steady-state non-photochemical quenching in light. **(F)** *Fv/Fm_Lss*, the PSII quantum yield of light adapted sample at steady-state, respectively. Quantum yield is the indicator of photosynthetic efficiency. For all the panels above, n = 5 plants. Differences are significant at $P < 0.05$ (*) and $P < 0.01$ (**) by Student's t-test.

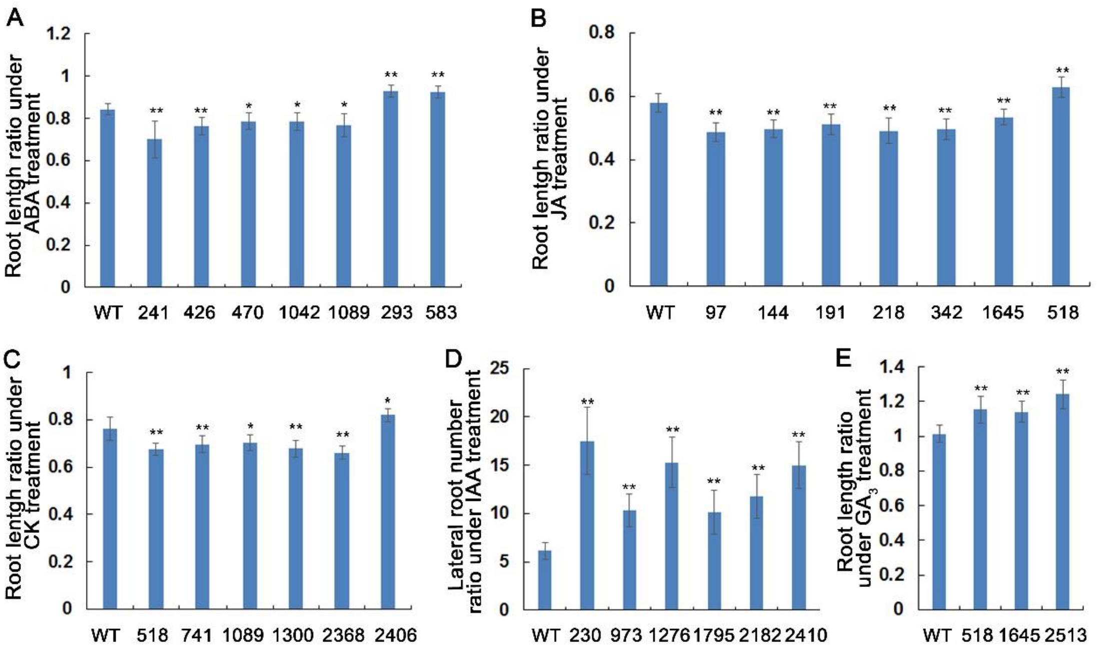

Supplementary Figure 6. amiRNA response to plant hormone treatments. Shown are amiRNA lines presenting a significant phenotypic response to different plant hormones: abscisic acid (trans-ABA), jasmonic acid (JA), cytokinin (t-Zeatin, CK), auxin (IAA), and gibberellin (GA_3). **(A)** Lines showing hypersensitivity or reduced sensitivity of root elongation to 1 μM ABA treatment. **(B)** Lines showing hypersensitivity or reduced sensitivity of root elongation to 50 μM JA treatment. **(C)** Lines showing hypersensitivity or reduced sensitivity of root elongation to 1 μM t-Zeatin (CK) treatment. **(D)** Lines of lateral roots highly induced with 250 nM IAA application. **(E)** Root elongation of lines showing hypersensitivity to 5 μM GA treatment. For all the panels above, n = 20 plants. Differences are significant at $P < 0.05$ (*) and $P < 0.01$ (**) by Student's t-test.

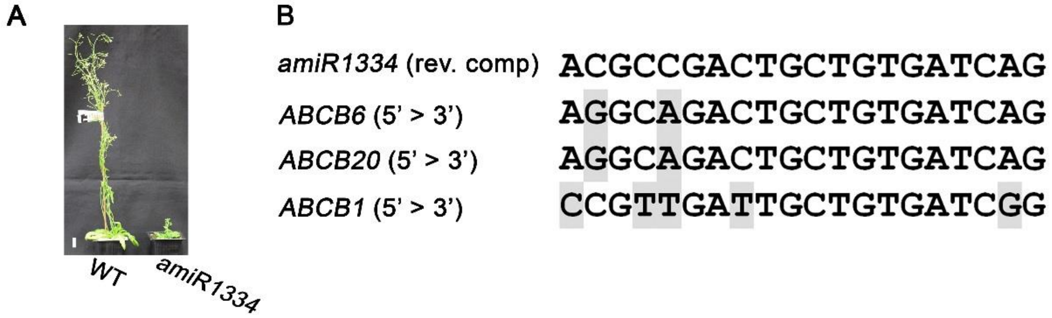

Supplementary Figure 7. Phenotype and target sequence of *amiR1334*. (A) Shoot phenotype of mature WT and *amiR1334* plants. Scale bar, 1 cm. **(B)** *amiR1334* sequence aligned to *ABCB6*, *ABCB20* and *ABCB1* recognition sites. Gray shading indicates mismatches.

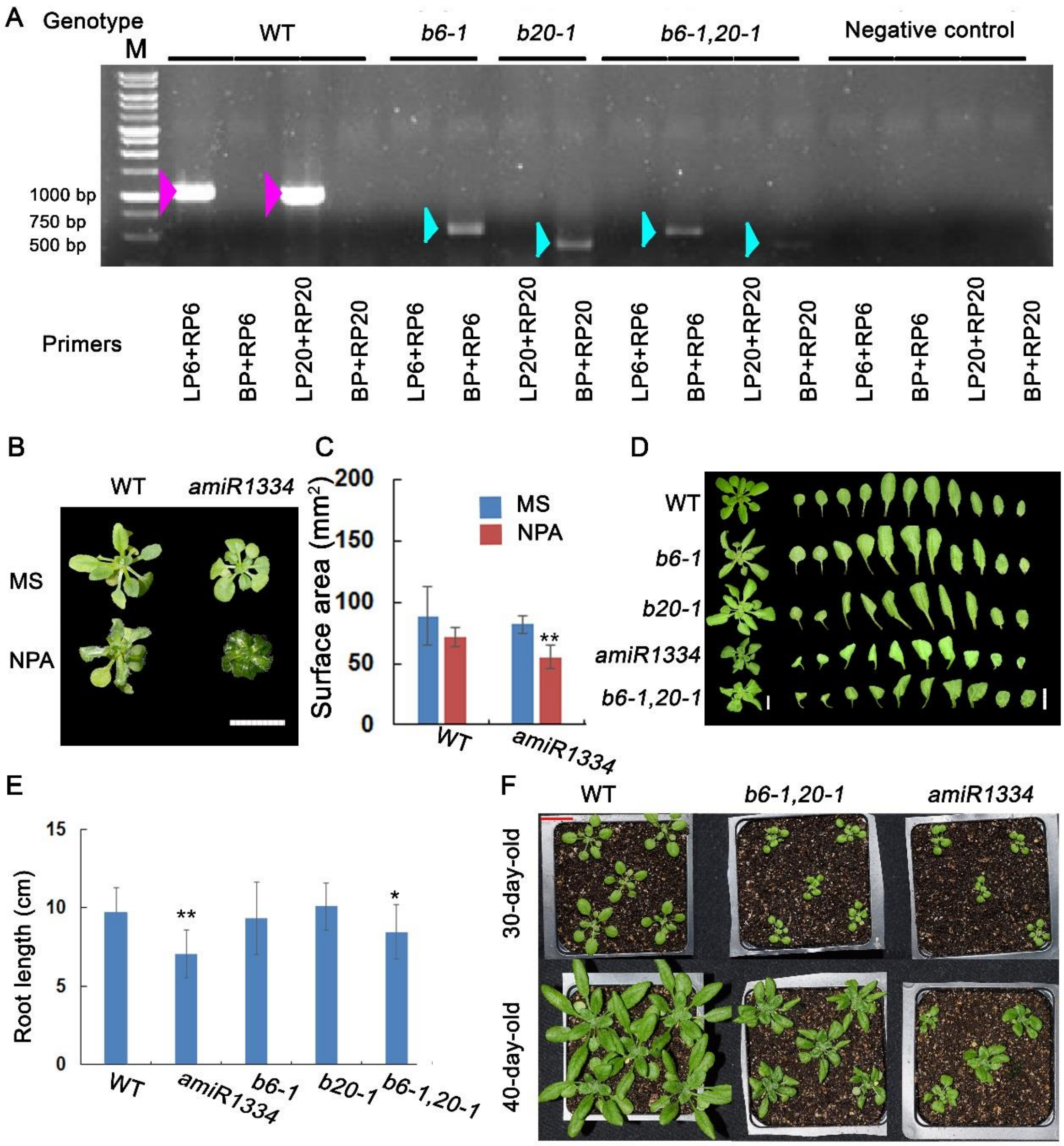

Supplementary Figure 8. Characterization of *amiR1334* and the *abcb6-1 abcb20-1* mutants. **(A)** Genotyping of the *abcb6-1* and *abcb20-1* single and double mutant plants. LP6 and RP6 (left primer and right primer for *ABCB6*). BP (T-DNA insertion primer). LP20 and RP20 (left primer and right primer for *ABCB20*). Magenta arrows indicate WT amplification bands. Turquoise arrows indicate T-DNA insertion amplification band. **(B)** Shoot phenotypes of three-week-old plants grown on ½ MS for one week and transferred to ½ MS with 5 μM NPA for two weeks. Scale bar, 1 cm. **(C)** Leaves surface area of the indicated genotypes described in (B). Shown are averages (±SD), * indicates for statistically significant differences relative to WT at $P < 0.05$ by student t-test at $P < 0.05$. n = 4 plants. **(D)** Three weeks old plants (left), and leaf gradient (right) of the indicated genotypes. Scale bar, 1 cm. **(E)** Root length of 7-day-old seedlings. Shown are averages (±SD), * indicate statistically significant differences relative to WT at $P < 0.05$ by student t-test at $P < 0.05$. n = 20 plants. **(F)** Phenotypes of 30-day-old and 40-day-old seedlings of *amiR1334* and *b6-1,20-1* plants. *b6-1,20-1* stands for *abcb6-1 abcb20-1* double mutant. Scale bar, 1 cm.

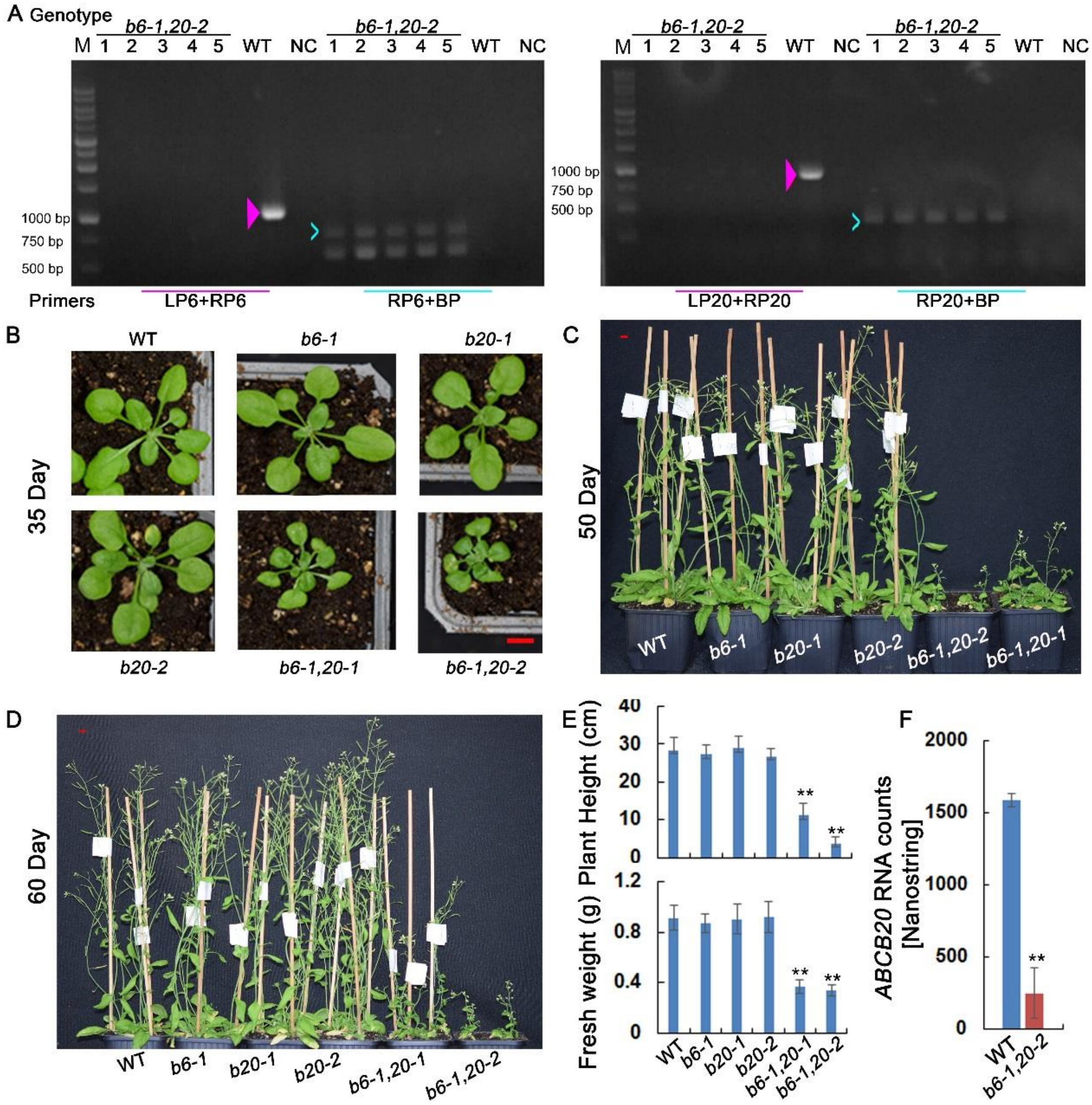

Supplementary Figure 9. Characterization of the *abcb6-1,20-2* double mutants. (A) Genotyping of the *abcb6-1 abcb20*-2 double mutant plants. LP6 and RP6 (left primer and right primer for *ABCB6*). BP (T-DNA insertion primer). LP20 and RP20 (left primer and right primer for *ABCB20*). Magenta arrows indicate WT amplification bands. Turquoise arrows indicate T-DNA insertion amplification band. **(B-D)** Phenotypes of 35, 50, 60-day-old seedlings of the indicated genotypes. Scale bar, 1 cm. **(E)** Plant height and fresh weight quantification of mature plants. *b6-1,20-1* stands for *abcb6-1 abcb20-1* double mutant, *b6-1,20-2* stands for *abcb6-1 abcb20-2* double mutant. Differences are significant at P < 0.01 (**) by Student's t-test. n = 10 plants. **(F)** *ABCB20* expression level in WT and *b6-1,20-2*. *b6-1,20-2* stands for *abcb6-1 abcb20-2* double mutant. Tissue was collected from plant inflorescence. Differences are significant at P < 0.01 (**) by Student's t-test. n = 3 biological replicates.

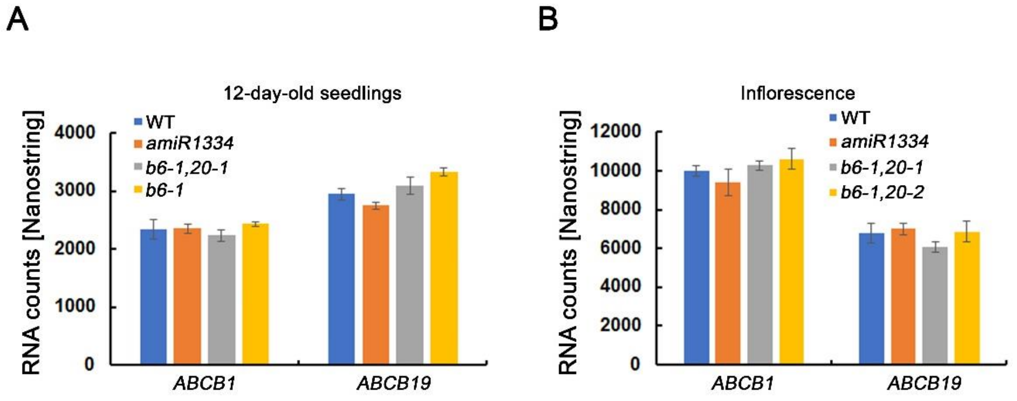

Supplementary Figure 10. ***ABCB1*** **and** ***ABCB19*** **are not down regulated in** ***abcb6*** **and** ***abcb20*** **loss-of-function lines compared to WT**. Nanostring RNA counts for the indicated genotypes. Tissue was collected from: **(A)** Twelve-day-old seedlings, **(B)** Inflorescence. Results are not significant at P < 0.01 by Student's t-test. n = 3 biological replicates.

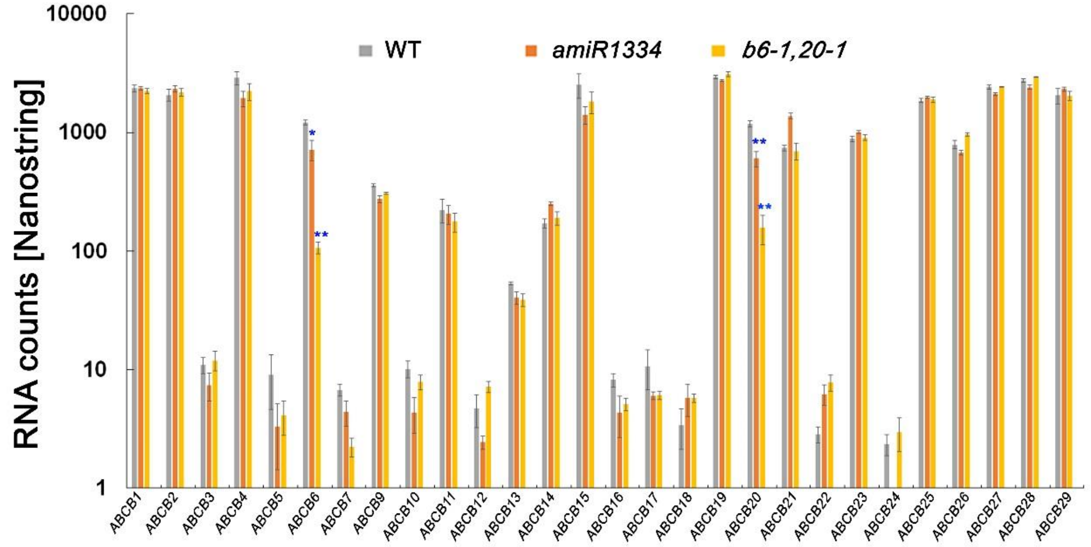

Supplementary Figure 11 Expression level of all ABCBs in 12-day-old WT, ***amiR1334*** **and** ***b6-1,20-1*** **seedlings.** Blue asterisk indicates for *ABCBs* which are significantly different from both *amiR1334* and *b6-1,20-1* compared to WT. Differences are significant at P < 0.05 (*) and P < 0.01 (**) by Student's t-test. n = 3 biological replicates.

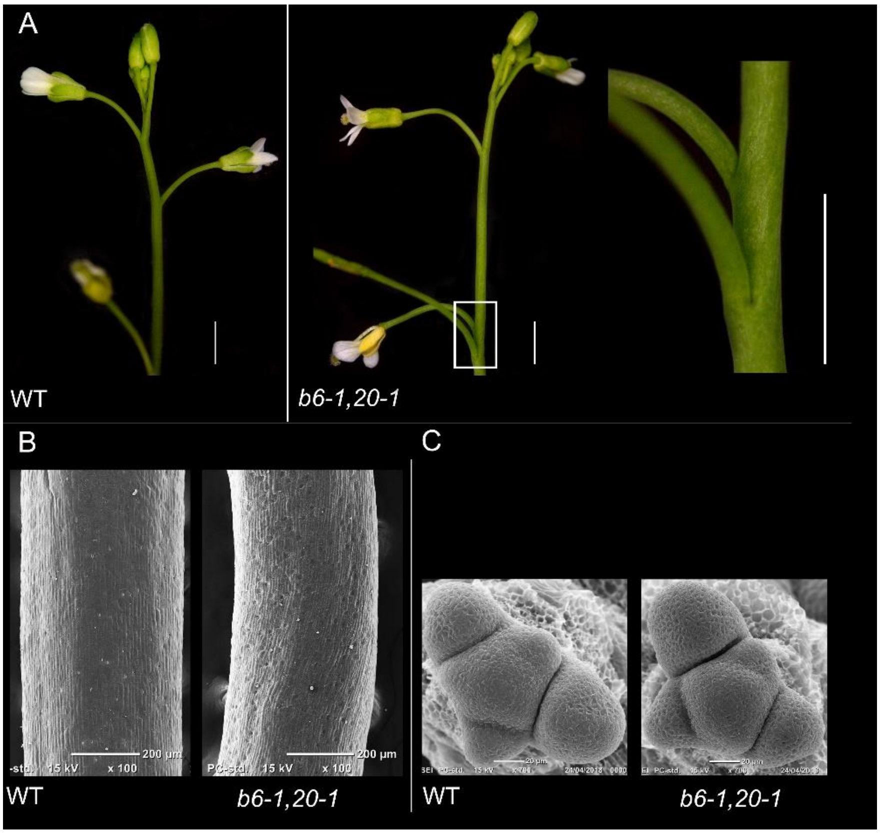

Supplementary Figure 12. *abcb6-1 abcb20-1* double mutant shows defects in the lateral organ spacing and stem twisting. (A) Spacing between siliques initiation sites in *b6-1,b20-1* compared to WT. The right image is a magnification of the white box area (middle image). Scale bar, 1 mm. **(B-C)** SEM images for *b6-1,20-1* double mutant stem twisting (B) and inflorescence meristem (C) compared to WT. Scale bar, 200 µm. *b6-1,20-1* stands for *abcb6-1 abcb20-1* double mutant.

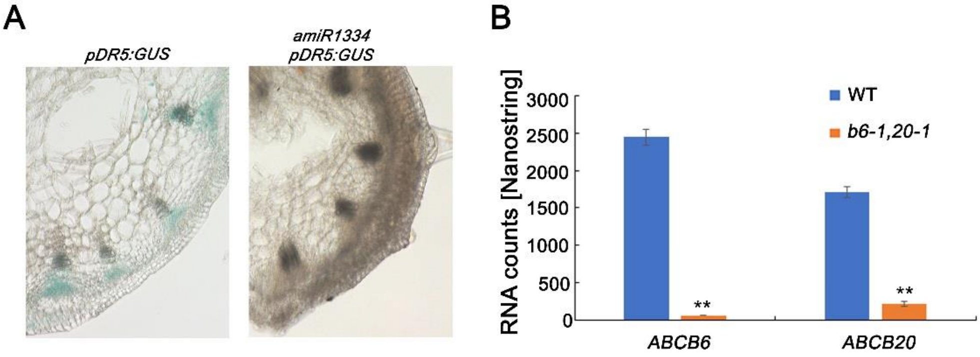

Supplementary Figure 13. *ABCB6* and *ABCB20* expression level and *pDR5:GUS* activity are down-regulated in *b6-1,20-1* compared to WT stem sections. (A) Shown are basal side stem sections. *pDR5:GUS* reporter activity was imaged in response to IAA application to the apical side of the segment. *amiR1334* shows reduction of *pDR5:GUS* activity at the basal side. See figure 4A-B for comprehensive description of the experiment. **(B)** Nanostring RNA counts on stem sections for the indicated genotypes. Differences are significant at $P < 0.01$ (**) by Student's t-test. n = 3 biological replicates. *b6-1,20-1* stands for *abcb6-1 abcb20-1* double mutant.

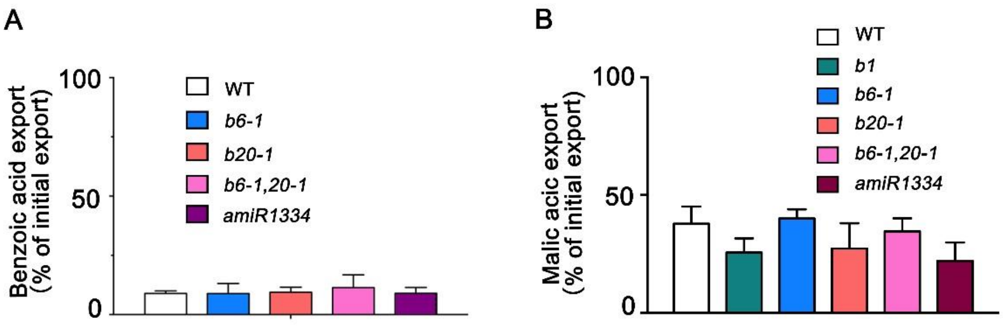

Supplementary Figure 14. Benzoic acid and malic acid export assays. (A) [^{14}C]BA and **(B)** [^{14}C]Malic acid export assays from *Arabidopsis* leaf mesophyll protoplasts. Results are not significant for P < 0.05, Welch's *t-test*. n = 4. *b6-1* and *b20-1* stands for *abcb6-1* and *abcb20-1* single mutant, respectively. *b6-1,20-1* stands for *abcb6-1 abcb20-1* double mutant.

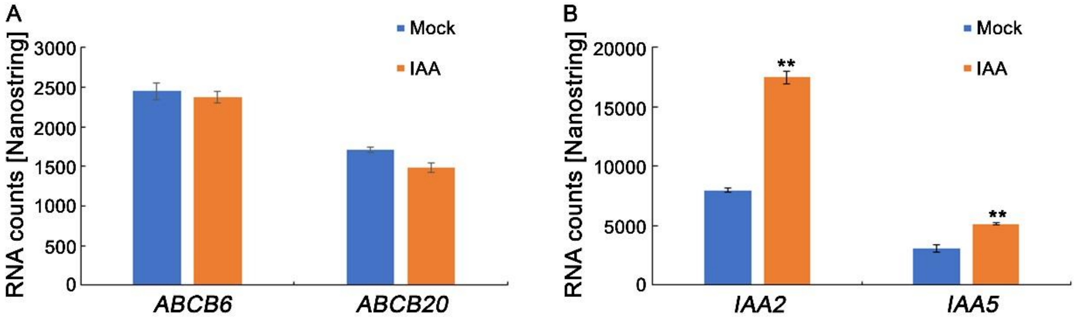

Supplementary Figure 15. ***ABCB6*** **and** ***ABCB20*** **do not respond transcriptionally to auxin treatment. (A)** *ABCB6* and *ABCB20* expression levels with and without auxin treatments. **(B)** Positive controls auxin response genes *IAA2* and *IAA5* expression levels with and without auxin treatments. The experiment was carried on WT inflorescence stem with and without 5 µM IAA treatment. n = 3 biological replicates.

Supplementary Table 1: Summary of significant amiRNA lines phenotypes and hormone response. Abbreviations: Cytokinin (C), IAA (I), ABA (A), Gibberellin (G), Jasmonic Acid (J), data presented in Supplementary Figure 6 and Fig. 2. Summary of plant Height (H). Morphological parameters presented in Supplementary Figure 4 and Fig. 1. (M). Photosynthesis parameters presented in Supplementary Figure 5 and Fig. 1. (P).

amiRNA #	Gene ID	Classification	C	I	A	G	J	H	M	P
98	AT1G59870, AT2G26910, AT3G16340	Plant primary active transport, ABC group (ATP-binding ATPase).						√	√	√
192	AT2G37360, AT5G06530							√	√	√
426	AT1G15520, AT5G58270				√			√	√	√
776	AT1G66950, AT3G30842, AT4G15233, AT4G15236								√	√
1234	AT2G47800, AT3G62700							√		√
1334	AT2G36910, AT2G39480, AT3G55320			√				√	√	
2396	AT3G47740, AT5G61700							√	√	√
32	AT2G39510,AT3G56620	Plant carrier-mediated transport, DMT group (drug/metabolite transporter).							√	√
174	AT1G76670, AT4G09810									√
180	AT1G21070, AT5G42420							√	√	√
213	AT4G18195, AT4G18197, AT4G18205								√	√
260	AT3G17430, AT4G32390, AT5G25400								√	√
293	AT1G21890, AT2G40900				√					√
546	AT1G01070, AT1G11450, AT4G01450							√		√
649	AT1G44750, AT4G18210, AT4G18220							√		
741	AT1G34020, AT4G09810		√					√		
837	AT4G18190, AT4G18220							√		
855	AT1G43310, AT5G33320							√		√
1042	AT2G33750, AT4G18195				√			√		
1276	AT1G53660, AT3G14410			√						√
333	AT5G13740,AT5G13750	Plant carrier-mediated transport, MFS group (major facilitator superfamily).								√
342	AT2G22730, AT5G65687						√		√	
572	AT2G32830, AT5G43350, AT5G43360, AT5G43370							√	√	√
583	AT1G34580, AT4G02050				√					√
722	AT3G54700, AT5G43360							√	√	√
1321	AT1G07340, AT1G50310, AT3G19940, AT5G61520							√	√	√
1795	AT3G03090, AT5G13740			√				√	√	√
2368	AT1G15500, AT1G80300		√					√	√	√
97	AT1G73590, AT1G77110	Plant carrier-mediated transport, other groups.					√	√	√	√
218	AT1G73700, AT2G34360						√	√		
556	AT4G19960, AT4G33530, AT5G14880								√	
704	AT1G60160, AT3G02050, AT4G13420							√	√	
2918	AT1G73590, AT2G01420							√		
157	AT5G23810, AT5G49630	Plant carrier-mediated transport, APC group (amino acid/polyamine/organo -cation).						√	√	√
574	AT1G80510, AT3G30390, AT5G38820							√	7 4	√
979	AT1G24400, AT1G61270, AT1G67640, AT1G71680, AT3G01760							√	√	
2935	AT1G79900, AT3G21390, AT5G48970							√		
2985	AT3G19553, AT5G36940							√		√
165	AT1G16310, AT1G79520, AT2G39450	Plant carrier-mediated transport, other cation carrier groups (CPA, CaCA, CDF, CaCA2).						√	√	√
191	AT1G05580, AT3G17630						√	√		√
510	AT1G05580, AT5G58460								√	√
518	AT1G06970, AT1G79610, AT2G30240		√			√	√	√	√	√

1645	AT3G05030,AT3G06370,AT5G55470					√	√	√		√
34	AT2G46320, AT4G27940	Plant carrier-mediated transport, Mitochondrial carrier (MC).						√	√	√
973/2410/2182	AT3G51870, AT5G01500			√				√	√	√
2406	AT4G26180, AT5G07320, AT5G61810		√							
141	AT3G07520, AT5G48400, AT5G48400	Plant channel mediated transport, channels.						√	√	√
226	AT4G18290, AT4G32650							√	√	√
230	AT3G24300, AT4G28700			√				√		√
470	AT1G80760, AT4G10380				√				√	√
698	AT3G11680, AT3G18440								√	√
998	AT3G16240, AT3G47440									√
2006	AT2G45960, AT4G00430, AT4G17340									
2867	AT2G16850, AT2G37180, AT2G39010, AT3G53420, AT3G54820, AT3G61430, AT4G35100									
2875	AT2G26975, AT3G46900, AT5G59030							√	√	
2457	AT3G01280, AT3G49920, AT5G15090, AT5G67500	Plant channel mediated transport, porins.						√	√	√
1200	AT1G13210, AT3G25610	Plant primary active transport, p-type ATPase.						√		√
1385	AT1G07670, AT1G17260, AT2G07560, AT2G18960							√	√	√
241	AT2G07671,ATMG00040	Others			√				√	√
474	AT1G10390,AT1G59660							√		√
652	AT3G07730, AT4G01170							√	√	√
686	AT1G72160, AT4G09160									√
735	AT1G35720, AT5G10220, AT5G10230, AT5G65020									
846/1049	AT1G65950, AT2G39190, AT4G24810, AT4G31390, AT5G50330							√	√	√
941	AT1G08980, AT3G17970, AT5G09420							√		
1089	AT3G09030, AT4G30940		√		√				√	
1162	AT1G22530, AT1G72150, AT1G72160, AT4G09160							√	√	
1300	AT2G16650, AT5G60430		√					√		
1345/1496	AT5G01040, AT5G01050, AT5G05390							√		√
2334	AT2G18180, AT2G21520, AT4G36490									√
2487	AT1G21850, AT4G12420, AT5G48450							√	√	√
2502	AT4G16143，AT5G03070							√		
2601	AT4G14160, AT5G43670									
2722	AT1G02690, AT1G09270, AT4G02150, AT4G16143							√		√
2889	AT1G11180, AT1G61250							√		√
95	Not sequenced								√	√
140								√	√	√
144							√	√	√	√
594										
779								√	√	√
1800										√
2495								√		√
2513						√		√	√	
2559										√
2625										
2691										
2694								√		√
2705								√		√
2725								√		√
2991								√		
2995								√		√

Supplementary Table 2. Genotyping primers for amiRNA lines (5'-3')

Primer name	Primer sequence
amiRNA_F-Forward Primer	CGTAAGGGATGACGCACAATC
amiRNA_R-Reverse Primer	ATGCGATCATAGGCGTCTCG

Supplementary Table 3. T-DNA lines and insertion IDs

Gene	Gene accession #	T-DNA line	Insertion
ABCB1	AT2G36910	SALK_083649	chr2 15504989
ABCB6	AT2G39480	GABI_401D12	chr2 16482050
ABCB20	AT3G55320	GABI_520G10	chr3 20510086
ABCB20	AT3G55320	GABI_387F09	chr3 20511364

Supplementary Table 4. Genotyping primers for T-DNA lines (5'-3')

**Gene**	**Primer name**	**Primer sequence**
abcb1	*abcb1*-LP	GAAGACTGCGACAAGGACAAG
	abcb1-RP	GCAAGAGCGATGTTGAAGAAC
	BP	ATTTTGCCGATTTCGGAAC
abcb6-1	*abcb6*-LP	ATATTGCATCGACTTGGATGC
	abcb6-RP	AGACTAAGCAAAGCAGGCTCC
	BP	ATATTGACCATCATACTCATTGC
abcb20-1	*abcb20-1*-LP	AGCCACTCTGGGAAACTAAGC
	abcb20-1-RP	TTTCTTCTAGGTCGGGAGAGC
	BP	ATATTGACCATCATACTCATTGC
abcb20-2	*abcb20-2*-LP	AACCCTATTTGGCTTCTCAGC
	abcb20-2-RP	ATTTTCTAGTGCTCCCTTCCG
	BP	ATATTGACCATCATACTCATTGC

Supplementary Table 5. Cloning primers (5'-3')

**Promoter/gene**	**Forward primer**	**Reverse primer**
pABCB6	CACCTTCGGCTTGGAATGGAAAC	AGCTACGACGGGGGGGAATTATATC
pABCB20	AAATACTGTAATCAAGTTTGCTTTG	CTTCGGAGGAGCACC
ABCB6 CDS	CACCATGATGATTTCGAGAGGTTTGTT TGG	GGGGACAAGTTTGTACAAAAAAGC AGGCTTAATGATGATTTCGAGAGGTTTGT

Supplementary Table 6. Nanostring Probes

Customer Identifier	Accession	Position	Target Sequence
ABCB1	NM_129247.3	1303-1402	GCTTTGGTACGGTGGTTATCTAGTTCGCCACCATTTGACCAACGGTGGTCTCGCTATTGCCACAATGTTCGCCGTTATGATTGGTGGATTGGCATTGGGA
ABCB2	NM_118729.4	1990-2089	TCGTCTCCAAGAGACTGCCTCATTGCAGCGTAACCCTTCCTTGAATCGCACGCTAAGCAGACCACATAGTATAAAATACTCGCGGGAACTATCAAGAACA
ABCB3	NM_116412.2	1671-1770	ATTGCTGTGATTCACCGCGGCAAGATAGTGGAAGAAGGTTCACACTCGGAGCTACTCAAGGACCATGAAGGGGCTTATGCACAACTTATACGGTTACAAA
ABCB4	NM_130268.4	3253-3352	ATCGAGTTCGTTATCTCCAGATTCCAGCAAAGCCGATGTAGCTGCTGCCTCGATTTTCGCAATAATGGACAGGGAATCAAAGATTGACCCAAGTGTGGAA
ABCB5	NM_116413.2	2315-2414	CAGCCTTGATCCGGACTCTTGTGGGAGACTCGCTTTGTCTATCGGTTAAAAACGTTGCGTCTTTGGTGACAGGACTAATCATAGCTTTCACGGCGAGCTG
ABCB6	NM_129506.2	2775-2874	GTTATTGCATTAGTAGTGACGACATACTATACGAGCAAGGGAAGCCACCTGCGTGAAGAGGTTGACAAGTGGTGTTTGATCATTGCCTGCATGGGAATAG
ABCB7	NM_124024.2	1973-2072	TCGGATAGCCAAAATGGAATCCATTCCGGCACTCTCACATCACCATCTGGTTTACCGGGAGTGATTAGCTTGGACCAGACGGAAGAGTTCCACGAGAATA
ABCB9	NM_117915.7	1815-1914	ACCTGAAACAAGCTTAGACGTCGAGAGGTCAGGAAGTCTTAGGCTATCATCTGCGATGAGAAGATCCGTTAGCCGGAATTCATCGAGCAGCCGCCATTCT
ABCB10	NM_100944.2	1969-2068	CTCCAAGCTTACCAGTCAGTACAAAACCCTTGCCGGAACTACCAATCACGGAGACGACCTCGTCCATTCATCAATCAGTAAACCAACCAGATACGACCAA
ABCB11	NM_100133.3	2074-2173	AAAAGTCAATGGAGGGAACTTCGTCGGTTGGTAATAGTAGCCGTCATCATTCTTTGAATGTTCTTGGTTTAACCACAGGACTAGACCTCGGCAGCCATAG
ABCB12	NM_100134.4	2070-2169	TTATCAGTGGTGGAACCTCGTCTTTTGGAAATAGCAGTCGTCATCATTCTTTGAATGTCCTTGGTTTATTCGCTGGACTAGACCTTGGTAGCGGTAGCCA
ABCB13	NM_102559.2	2167-2266	GTGCAGTACTTGCTGGAGCACAAACTCCATTGTTCTCTATGGGGATTGCTTATGTGTTAACTGCATTTTATTCGCCTTTTCCTAATGTGATCAAGCGCGA
ABCB14	NM_102566.3	3011-3110	GGTATATCTCAGTTCTGATTAAGCGCAACGAGACCAACTTTGAGGATAGTATCAAATCTTTCATGGTATTGCTCGTCACTGCTTACTCCGTAGCGGAAAC
ABCB15	NM_113754.3	2109-2208	GGATCCGAGCAAAGATATAAGAAATAGTTCTAGAGTGTCGACTCTTAGCCGTTCAAGCTCTGCTAACTCGGTTACAGGTCCAAGCACTATAAAGAATCTT
ABCB16	NM_113756.5	2634-2733	ACAAGAACGGATCATGAAATTACTCGAGAGAGTTCAAGAAGGTCCACGAAGAGAAAGTGCCCGCCAATCATGGTTAGCTGGGATTATGTTGGGGACTACG
ABCB17	NM_113758.2	2520-2619	GAGCTTGTCCGAAAAGGCAAGCAAAGCCCAAGATGAAAGTAGTAAATTAGCGGCAGAGGCGGTATCAAACATACGAACAATCACTGCCTTCTCGTCACAA
ABCB18	NM_113759.1	930-1029	CTCAGAGGCGTTTGTCGTAGGAGAACGGATTATGAAAGTGATAAATAGAGTCCCTGGTATAGACTCAGACAACTTGGAAGGTCAGATACTTGAGAAAACT
ABCB19	NM_113807.3	2780-2879	TGGAGAGTCTCACTTCTCATCTTAGGCACATTCCCACTTCTAGTCCTCGCTAACTTTGCTCAGCAACTATCTCTGAAGGGTTTTGCTGGAGACACAGCTA
ABCB20	NM_115390.3	2479-2578	TGACATCAGATCCTAAAAACGAGCGTTCCCATTCGCAGACGTTTAGTCGTCCTCTTAGTTCTCCTGATGACACTAAAGCAAATGGTAAGGCGTCTAAGGA
ABCB21	NM_116080.3	1591-1690	TAATGTGAACTTTAGTTACCCGGCAAGACCAGAAGAGCAGATCTTTCGCGGGTTTTCACTCTCTATCTCGAGTGGTTCAACCGTGGCTCTAGTTGGGCAG
ABCB22	NM_148757.1	2284-2383	GACGCAAACGTGGTGAGATCGCTTGTTGGTGAACGAGTGTCGTTGTTGGTACAAACTATATCAGCAGTGAGTGTAGCTTGCACACTTGGTTTGGCCATTT
ABCB23	NM_119005.3	137-236	GTGATTCGTTCGGACAAGAAGCGATGATGAGGGGATCTCGGTTTCTCCTCTCTCGCGCTTCTCTGTATCACCGTCTTCGCTCTGATCATCACCATCACAG
ABCB24	NM_119004.1	549-648	AGCTCTCCGCACCATACGAACCATCTCTAGAAAAGTTCTCTCGCGTTTACACGATCTCGATCTTCGTTACCATCTAAATAGAGACACAGGTGCTTTGAAC
ABCB25	NM_125212.3	1767-1866	GGAATTTCCTTTGTCGTACCGGCAGGAAAAAGTGTGGCAATCGTTGGTACTAGCGGGAGCGGCAAGTCAACAATTCTTAGGATGCTATTCAGATTCTTCG
ABCB26	NM_105729.6	1300-1399	TGCAACTCAGATTATTGCTGTCCTGGTTGGAGGATTGTCTATCTTAGCTGGTCAAATAACAGCAGAGCAGCTGACAAAGTTTCTGTTGTATAGTGAGTGG
ABCB27	NM_123266.8	1271-1370	CACAACTGCAATGAAAGCTGCAGGCGCTAGTAGACGAGTTTTTCAGATCTTGGACCGTGTTTCTAGCATGTCAAGTTCAGGGGATAAGTGTCCAGTCGGT
ABCB28	NM_118677.5	1313-1412	TGTCCAAGGGCTTGTAAACACGTTTGGAGATCTACGTGGGACTTTTGCTGCTATTGACAGGATCAATTCCATTTTAAACGCGGTGGACATAGATGAGGCT
ABCB29	NM_120473.3	162-261	CATTTCAACCATCTCCGAGACCTTTGAGCTTCTGTAAACCCTCCGCTCTCCGTCTCCGAGCCAATACAACAGTCAATTCACTAAAAGCCCTCGAAACTAT
GH3.3	NM_127881.3	1353-1452	GAGGAAGAACGTTTTGCTTAGCATTGAGTCGGATAAAACCGATGAAGCTGAGCTCCAAAGCGCGGTTGAGAACGCATCGCTCTTACTTGGAGAGCAAGGA
IAA2	NM_113203.5	208-307	ACAGAGCTATGTCTTGGATTACCCGGAAGAACAGAGAAGATCAAAGAAGAACAAGAGGTTTCTTGCGTTAAAAGTAACAACAAGCGTCTATTTGAGGAAA
IAA5	NM_101427.4	164-263	GATTTGAAATGTGAACCGGCGAAAAAGAGTCAAGTTGTGGGTTGGCCACCGGTTTGTTCGTACCGGAGAAAGAACAGTCTCGAACGGACCAAAAGTTCGT
PP2A (housekeeping)	NM_101203.5	588-687	TTCAGCTGGTGAATGGTTCACAGCCAGAGTATCAGCATGTGGGATTTTCCATATTGCATACCCAAGTGCCCCAGATGTGCTAAAGACGGAGCTAAGATCA
ACT2 (housekeeping)	NM_112764.4	1474-1573	TGATGAGGCAGGTCCAGGAATCGTTCACAGAAAATGTTTCTAAGCTCTCAAGATCAAAGGCTTAAAAAGCTGGGGTTTTATGAATGGGATCAAAGTTTCT
CBP20 (housekeeping)	NM_123787.2	686-785	GCTTATCCTCAAGCTGCGCCAACGAATTATGGAAATGGAAGGCGTGGTGGTGGAAACTATGGTCAAGGAGGACAAAATCGCCATGGAAGAGGAGACTACC
